# Post-herpetic hypertrophic keloid: a rare clinical presentation

**DOI:** 10.11604/pamj.2025.52.132.49505

**Published:** 2025-11-28

**Authors:** Punam Gaurav Sawakar, Pawan Banduji Itankar

**Affiliations:** 1 Department of Panchakarma, Mahatma Gandhi Ayurved College Hospital and Research Centre, Datta Meghe Institute of Higher Education and Research, Wardha, Maharashtra, India,; 2 Department of Rachana Sharir, Mahatma Gandhi Ayurved College Hospital and Research Centre, Datta Meghe Institute of Higher Education and Research, Wardha, Maharashtra, India

**Keywords:** Keloid, herpes zoster, hypopigmentation

## Image in medicine

A 42-year-old female came to the Panchakarma outpatient department with complaints of a thickened scar over the right and left arm region with blackish discolouration, intermittent itching, pricking pain (especially in the rainy season) and mild serous discharge (on and off) for two years. She has a history of herpes zoster over the bilateral axillary and arm region two years ago. After treating the herpes zoster, within 12-15 days, keloid scar formation was obtained over multiple affected body regions. There is skin contracture and elevated skin thickening at borders with blackish discolouration over the lateral aspect of the right and left arm; based on this, a diagnosis of hypertrophic keloid was confirmed. The fibroproliferative wound healing response known as keloids is characterised by excessive and invasive growth of elevated scar tissue beyond the initial boundaries of the wound. Trauma, burns, surgeries, vaccinations, skin piercings, acne, and herpes zoster are common causes of injury and discomfort. Keloid incidence varies by ethnicity, with rates of 5-10% in Africa, 0-0.1% in Asia, and <0.1% elsewhere. Higher skin pigmentation is strongly linked to increased keloid risk. Superficial spreading keloids are primarily elevated at the borders and have a centrally located, flattened and quiescent area. While the borders exhibit hyperpigmentation, the middle region is typically hypopigmented. In contrast, elevated keloid scars may have small patches of core inactivity and have a pronouncedly bulbous appearance with clear borders.

**Figure 1:**
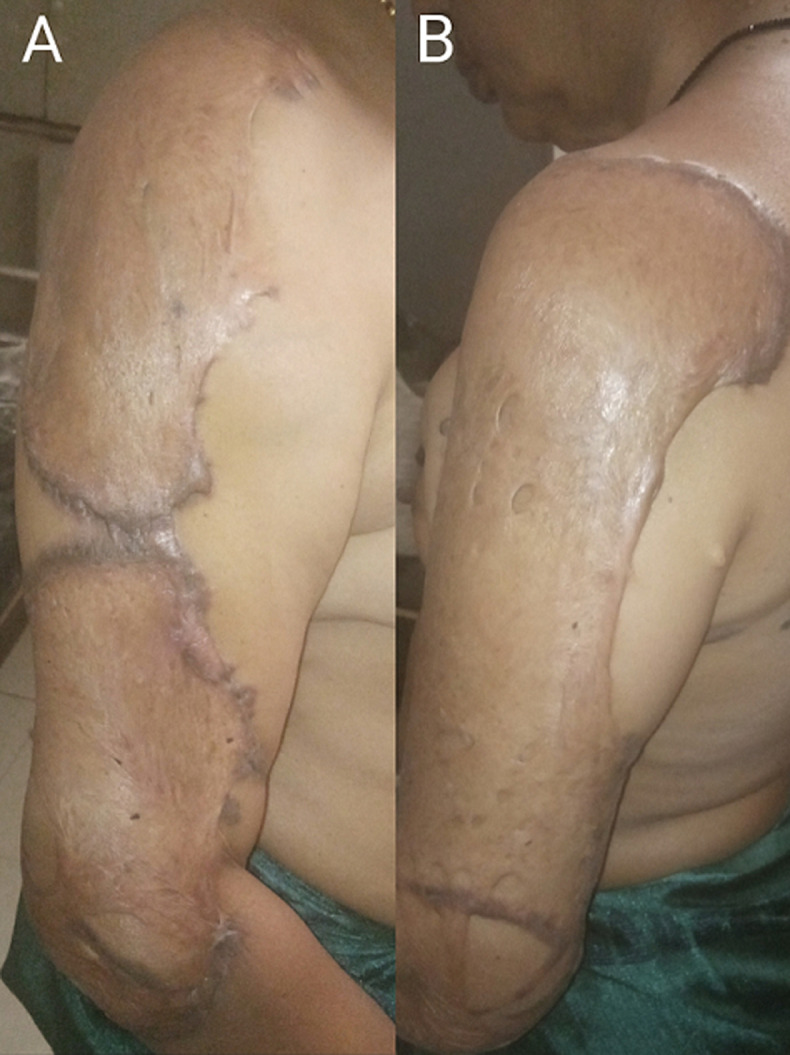
A, B) raised and thick hypertrophic keloid scars on the lateral aspect of the right arm, and raised skin thickening on the lateral aspect of the left arm

